# Temporal dynamics of immune response following prolonged myocardial ischemia/reperfusion with and without cyclosporine A

**DOI:** 10.1038/s41401-018-0197-1

**Published:** 2019-03-11

**Authors:** Vitali Rusinkevich, Yin Huang, Zhong-yan Chen, Wu Qiang, Yi-gang Wang, Yu-fang Shi, Huang-tian Yang

**Affiliations:** 1 CAS Key Laboratory of Tissue Microenvironment and Tumor, Laboratory of Molecular Cardiology, Shanghai Institute of Nutrition and Health, Shanghai Institutes for Biological Sciences, University of Chinese Academy of Sciences, Chinese Academy of Sciences, Shanghai, 200031 China; 2 CAS Key Laboratory of Tissue Microenvironment and Tumor, Laboratory of Tumor and Stem Cell, Shanghai Institute of Nutrition and Health, SIBS, University of Chinese Academy of Sciences, Chinese Academy of Sciences, Shanghai, 200031 China; 3 Department of Pathology and Laboratory Medicine, College of Medicine, University of Cincinnati Medical Center, Cincinnati, OH 45267 USA

**Keywords:** myocardial infarction, ischemia reperfusion, late reperfusion, immune response, inflammatory cytokines, angiogenesis, cyclosporine A

## Abstract

Understanding the dynamics of the immune response following late myocardial reperfusion is critical for the development of immunomodulatory therapy for myocardial infarction (MI). Cyclosporine A (CSA) possesses multiple therapeutic applications for MI, but its effects on the inflammation caused by acute MI are not clear. This study aimed to determine the dynamics of the immune response following myocardial ischemia/reperfusion (I/R) and the effects of CSA in a mouse model of prolonged myocardial ischemia designated to represent the human condition of late reperfusion. Adult C57BL/6 mice were subjected to 90 min of closed-chest myocardial I/R, which induced severe myocardial injury and excessive inflammation in the heart. Multicomponent analysis of the immune response caused by prolonged I/R revealed that the peak of cytokines/chemokines in the systemic circulation was synchronized with the maximal influx of neutrophils and T-cells in the heart 1 day after MI. The peak of cytokine/chemokine secretion in the infarcted heart coincided with the maximal macrophage and natural killer cell infiltration on day 3 after MI. The cellular composition of the mediastinal lymph nodes changed similarly to that of the infarcted hearts. CSA (10 mg/kg/day) given after prolonged I/R impaired heart function, enlarged the resulting scar, and reduced heart vascularization. It did not change the content of immune cells in hearts exposed to prolonged I/R, but the levels of MCP-1 and MIP-1α (hearts) and IL-12 (hearts and serum) were significantly reduced in the CSA-treated group in comparison to the untreated group, indicating alterations in immune cell function. Our findings provide new knowledge necessary for the development of immunomodulatory therapy targeting the immune response after prolonged myocardial ischemia/reperfusion.

## Introduction

Myocardial infarction (MI) is a leading cause of morbidity and mortality throughout the world. Coronary artery reperfusion therapy is one of the most successful therapies in modern medicine. Early reperfusion is obviously a preferred therapy for myocardial infarction. However, a high proportion of patients are admitted beyond the time window when successful rescue of the myocardium is possible [1, 2]. Kim and Braunwald [3] have proposed that late reperfusion – too late to reduce myocardial infarct size, but early enough to favorably affect infarct healing – also appears to limit infarct expansion and left ventricular (LV) remodeling (the open-artery hypothesis). Late reperfusion has shown its efficacy in both animal and human research [2–5]. However, the therapeutic potential of late reperfusion is significantly lower than that of early reperfusion. Therefore, understanding the pathophysiological basis of late reperfusion is a prerequisite for developing additional therapy for those patients.

Inflammation plays a critical role in the process of myocardial ischemia/reperfusion (I/R) injury and healing, as evidenced by experimental and clinical studies published over the past 20 years. The immune system is evolved to promote tissue homeostasis following tissue damage after MI [6–8], but a few findings support the case that the immune response to infarction is unnecessarily intense [9]. Increasing experimental evidence suggests that immune-regulating therapies along with reperfusion can improve healing after MI, while characterization of the immune response following various durations of ischemia is critical for the development of clinically accepted immune-modulating therapy for MI [10]. The dynamics of inflammation in permanent ligation and short I/R in mice have been reported [11], but the pattern of immune response following prolonged myocardial I/R remains unknown.

Cyclosporine A (CSA), extracted from the fungus *Tolypocladium*, is a potent suppressor of the immune system, particularly T-lymphocytes. The first use of CSA in cardiology was in heart transplantation as an immunosuppressive agent to suppress acute rejection and improve early graft survival. Similar to organ transplantation, nonautologous stem cell transplantation potentially requires host immunosuppression to improve the survival of transplanted cells [12]. Thus, CSA is given along with different types of stem cells in the acute phase of MI [13, 14]. Moreover, the discoveries of the mitochondrial permeability transition pore (MPTP) and the ability of CSA to regulate it have emerged as a promising strategy for cardioprotection [15]. As a result, CSA is postulated to prevent reperfusion injury in the heart through inhibition of MPTP opening, thus improving cardiomyocyte survival [16–18]. Nevertheless, despite the known immunosuppressive properties of cyclosporine and its wide application in different therapeutic approaches, both for heart protection and for heart repair, its direct effect on the postinfarction immune response is still unclear.

Animal models of MI have been employed in scientific practice to mimic human cardiac pathology. Therefore, the clinical condition of late reperfusion requires a representative animal model – a model of prolonged myocardial ischemia. Moreover, massive cardiomyocyte loss and reperfusion injury cause intensive inflammation after prolonged myocardial ischemia [19], making this model a useful tool not only to simulate human diseases but also to study the immune response after MI in relation to heart function and remodeling.

To address the above questions, the present study used a murine model of prolonged myocardial I/R designated to mimic the clinical condition of late reperfusion to determine (i) the functional and morphological characteristics of the reperfused murine heart after prolonged myocardial ischemia; (ii) the temporal dynamics of the immune response following prolonged myocardial I/R; and (iii) the effects of CSA on the immune response and cardiac recovery following prolonged myocardial I/R.

## Methods

### Animals

All experimental procedures on mice conformed to the Guidelines for the Care and Use of Laboratory Animals (NIH publication, 8^th^ edition, 2011) and were approved by the Institutional Review Board of the Shanghai Institutes for Biological Sciences, Chinese Academy of Sciences.

### Closed-chest myocardial I/R model

In an attempt to avoid any confounding influences of the excessive immune response associated with the classic open-chest model of I/R, we utilized a closed-chest model as described previously [20, 21]. Briefly, male C57BL/6 mice aged 12–14 weeks (The Shanghai Slac Laboratory Animal Co. Ltd, Shanghai, China) underwent surgical implantation of an occluding device around the left anterior descending coronary artery. Ten minutes prior to the initiation of surgery, the mice were injected with sodium pentobarbital (60 mg/kg; ip), and after orotracheal intubation, they were anesthetized using isoflurane (1.0 vol%). The mice were ventilated throughout the procedure with a stroke volume of 250 μL at a rate of 125 strokes/minute. The mice were subjected to a mini-thoracotomy in the fourth left intercostal space, after which an 8-0 polypropylene suture attached to a U-shaped tapered needle was passed under the proximal left anterior descending (LAD) artery ~2–3 mm from the tip of the left auricle. The needle was cut from the suture, and the two ends of the 8-0 suture were threaded through a 0.5 mm piece of PE-10 tubing (soaked for 24 h in 100% ethanol) before being exteriorized through the chest wall and tucked under the skin. The mouse was removed from the respirator and allowed to recover for 14 days before induction of infarction.

On the day the infarction was induced, the mice preimplanted with the occluding device were slightly preanesthetized with ketamine (100 mg/kg; ip), secured to a heated ECG board and further anesthetized with sevoflurane (1.0 vol%). The mice were breathing spontaneously throughout the experiment. The skin over the old scar was opened, and both ends of the LAD artery suture were taped to a 5 g lead weight. The lead weight was hung on the bearing, allowing permanent compression of the LAD. S-T segment elevation was visible on the ECG, indicating successful occlusion of the coronary artery; ischemia was continued for 30 or 90 min. To induce reperfusion, we cut the sutures close to the chest wall, releasing the tension.

The mice assigned to sham surgery received the same surgery with the same instrumentation as the mice assigned to I/R injury, except that the ends of the suture encircling the LAD were not pulled. Sham-treated mice had the same duration of anesthesia, ventilation, and surgical manipulations as mice subjected to I/R injury.

### Experimental protocol

Twenty-four hours after infarction induction, the mice were subjected to echocardiography and randomized, and the first intraperitoneal injection of either CSA or vehicle was given. Two experimental groups were defined accordingly: the CSA treatment group and the vehicle control group (VEH). CSA (Sandimmune Novartis, Basel, Switzerland) was used at a dose of 10 mg/kg/day, and cremophor EL (Sigma-Aldrich, St. Louis, USA) was used at 130 mg/kg/day as a vehicle. Both compounds were mixed with normal saline to a final volume of 0.1 mL prior to injection. Treatment was continued daily until day 5 after MI (5 injections in total). The CSA serum concentration on day 5 after MI was measured by ELISA.

### Assessment of infarct size

Infarct size was measured using Evans Blue and TTC (2,3,5-triphenyltetrazolium chloride) staining as described previously [22]. Briefly, following 24 h of reperfusion, the mice were deeply anesthetized with sodium pentobarbital (90 mg/kg; ip) and connected to a ventilator as described above. The abdominal cavity was exposed, and the diaphragm was penetrated and dissected to obtain visual access to the heart. The skin incision used for the ischemia protocol was opened again, and both ends of the suture connected to the occluding device were taped to a 5 g lead weight for LAD compression. Using a 30-gauge needle, 0.1 mL of 1% Evans Blue solution was infused via the femoral vein. Several minutes after injection, when the clear borderline of the area at risk (AAR) had appeared, the heart beating was stopped in diastole by direct injection of 2% potassium chloride into the right ventricle. Immediately after the heart stopped beating, it was removed and washed in cold phosphate-buffered saline (PBS). Next, the heart was filled with melted 1% agarose and placed on ice in a freezer for 15 min. Then, the heart was cut into 1 mm sections starting just above the LAD artery suture, yielding 4–5 sections per heart. Sections were incubated in 1% TTC solution for 15 min and fixed in formalin. Both sides of each section were pictured. Infarcted area (IA) and AAR were assessed at the mid-infarction level using ImageJ software (version 1.47).

### Histological examination and immunohistochemistry

Four weeks after MI, the mice were deeply anesthetized, and the heart was removed from the thoracic cavity as described above. The hearts were rinsed in PBS and stopped in diastole using potassium chloride-saturated phosphate-buffered saline, after which they were fixed in optimal cutting temperature (O.C.T.) compound (Sakura Finetek, Torrance, USA), frozen at −80 °C and sectioned transversely (4 μm thick) at 500 μm intervals beginning from the apex. Masson’s trichrome stain was used to analyze the scar area of the infarcted hearts. For each section of the LV, the residual myocardium and the scar area were measured using MIQuant software as previously described [23]. The size of the scar was expressed as a percentage of the LV area.

For immunohistochemistry, the slides from the mid-infarction level of each heart were washed with PBS, fixed in 4% paraformaldehyde, blocked and permeabilized with 2% normal goat serum and 0.3% Triton X-100 in PBS and incubated overnight at 4°C with anti-von Willebrand factor (vWF; 1:400, Abcam, Cambridge, UK) and anti-α-smooth muscle actin (1:400, α-SMA; Sigma-Aldrich, St. Louis, USA) primary antibodies. The sections were then washed and incubated with the relevant fluorophore conjugated secondary antibodies, namely, goat anti-rabbit secondary antibody conjugated to Alexa Fluor 488 (1:1000, Invitrogen, Carlsbad, USA) and anti-mouse antibody conjugated to DyLight 549 (1:1000, Jackson Laboratory, Bar Harbor, USA), while Hoechst (1:2000) was used to identify nuclei. Fluorescent imaging was performed with an Axio Imager A2 microscope (Zeiss, Oberkochen, Germany). Images of the infarcted myocardium were obtained at ×20 magnification from 3 sections per heart, with a minimum of 15 fields of view from infarcted and border zones and a minimum of 9 fields from remote myocardium. The image analysis was performed semiautomatically using AngioTool 64 0.6a software. The cardiac vessel density was quantified as the ratio of either vWF- or α-SMA-positive vessel area to the total area of tissue per field.

### Echocardiography

Echocardiography was performed using an MS-400 imaging transducer with a digital ultrasound system, the Vevo 2100 Imaging System (Visual Sonics, Toronto, Canada). Mice were preanesthetized in an induction chamber using isoflurane and then transferred to a heated ECG platform for heart rate monitoring during the imaging procedure. The body temperature of the animals was maintained at 37 °C. During imaging, anesthesia was maintained with 1.0 vol% isoflurane delivered via a nosecone. Image acquisition was initiated with the transducer probe placed along the left sternal border to obtain the parasternal long-axis view, which displays both the apex and the outflow tract of the LV. An optimal parasternal long-axis (LAX) projection (i.e., visualization of both the mitral and aortic valves and maximum distance between the aortic valve and the cardiac apex) was used for orientation. LV dimensions and contractility were measured from short-axis M-mode images for at least 3 consecutive cardiac cycles and then averaged. M-mode-based measurements included LV end-systolic (LVESV) and end-diastolic volumes (LVEDV), LV ejection fraction (LVEF), and LV fractional shortening (LVFS).

### Isolation of single cells from the mouse heart and mediastinal lymph nodes

Mice were intracardially perfused with 50 mL of ice-cold HBSS with heparin to exclude blood cells. For heart digestion, we used a previously described protocol [24]. Briefly, the LV was dissected, minced with fine scissors, and enzymatically digested with a cocktail of type II collagenase (Worthington Laboratories, Worthington, USA) and collagenase/dispase (Roche Diagnostics, Risch-Rotkreuz, Switzerland) solution at 37 °C with gentle agitation. The cells were then passed through a 40 μm nylon mesh (BD Falcon, Franklin Lakes, USA), centrifuged (10 min, 500 *g*, 4 °C), resuspended in red cell lysis buffer (eBioscience, Santa Clara, USA) and incubated for 10 min. Next, the cell suspension was reconstituted with staining buffer (dPBS with no Ca^2+^ or Mg^2+^, 2% FBS). Mediastinal lymph nodes (MLNs) were isolated, homogenized, and suspended in PBS, and then passed through a 40-μm nylon mesh to remove connective tissue. The cells from digested hearts and MLNs were counted on a Countstar automated cell counter (Rui Yu Biotech, Shanghai, China).

### Flow cytometric analysis

Cell suspensions isolated from MLNs and hearts were subjected to flow cytometry as reported previously [11]. As a way of preventing additional bias in the results caused by variability in the manipulation, heart digestion and flow cytometry of cell suspensions obtained from murine hearts and MLNs were performed on the same day for the compared time points. To block nonspecific binding of antibodies to Fcγ receptors, we first incubated the isolated cells with anti-CD16/32 antibodies (BD Bioscience, Franklin Lakes, USA) at 4 °C for 5 min. Subsequently, the cells were stained with a mixture of antibodies at 4 °C for 20 min.

For flow cytometry, FITC-conjugated rat anti-mouse CD45, PE-Cy™7-conjugated rat anti-mouse CD11b, PE-conjugated rat anti-mouse F4/80, Alexa Fluor® 647-conjugated rat anti-mouse CD206, PE-conjugated rat anti-mouse Ly-6G, PerCP-Cy™5.5-conjugated rat anti-mouse Ly-6C, BV510-conjugated hamster anti-mouse CD11c, APC-conjugated mouse anti-mouse NK-1.1, PE-conjugated rat anti-mouse CD8a, BV510-conjugated hamster anti-mouse CD3e, PE-conjugated hamster anti-mouse CD3e, PerCP-Cy™5.5-conjugated rat anti-mouse CD4, PerCP-Cy™5.5-conjugated rat anti-mouse CD45R/B220, PE-Cy™7-conjugated rat anti-mouse IFN-γ, APC-conjugated rat anti-mouse IL-4, PE-conjugated rat anti-mouse IL-17A, APC-conjugated rat anti-mouse CD25, purified rat anti-mouse CD16/CD32, and PE-conjugated rat anti-mouse I-A/I-E (anti-MHC-II) antibodies were purchased from BD Pharmingen (San Diego, USA); PE-conjugated anti-mouse Foxp3 antibody was purchased from eBioscience (Santa Clara, USA). For intracellular cytokine staining, the cells were restimulated for 5 h in vitro in the presence of 1 μg/mL ionomycin (Sigma-Aldrich, St. Louis, USA), 50 ng/mL phorbol 12-myristate 13-acetate (Sigma-Aldrich, St. Louis, USA) and monensin 10 μg/mL (BD Biosciences, Franklin Lakes, USA). Flow cytometric analysis was performed on a Gallios flow cytometer (Beckman Coulter, Pasadena, USA) and analyzed using FlowJo x10.0.7r2 software.

### Protein preparation

Left ventricles were homogenized, and the cells were lysed as described previously [25]. Briefly, freeze-clamped LV tissues (~100 mg) were homogenized at 4 °C with a homogenizer in 10 volumes of lysis buffer containing 1% Triton X-100, 0.5% deoxycholate, and 5 mM 2-mercaptoethanol. Cell extracts were scraped into lysis buffer containing 20 mM Tris-HCl (pH 7.4), 6 mM urea, and 200 mM potassium chloride with a protease inhibitor cocktail of 3.6 mM leupeptin, 2.1 mM pepstatin A, and 50 mmol/l phenylmethylsulfonylfluoride, followed by vigorous vortexing and cooling on ice for 15 min and then 15 min of centrifugation at 12,000 × *g*. The protein concentration was measured by the bicinchoninic acid assay (BCA) using the Pierce BCA Protein Assay Kit (Thermo Scientific, Rockford, USA).

### Cytokine analysis

The concentrations of cytokines and chemokines in the heart and serum were measured using Bio-Plex protein array systems (Bio-Rad, Hercules, USA) based on xMAP technology (Luminex, Austin, USA). Two Bio-Plex Mouse Cytokine multiplex panels were used in combination, simultaneously quantifying 23 proteins: interleukin (IL)-1α; IL-1β; IL-2; IL-3; IL-4; IL-5; IL-6; IL-9; IL-10; IL-12 p40; IL-12 p70; IL-13; IL-17; eotaxin; granulocyte colony stimulating factor (G-CSF); granulocyte-macrophage colony stimulating factor (GM-CSF); interferon (IFN)-γ; keratinocyte chemoattractant (KC); monocyte chemoattractant protein (MCP)-1; macrophage inflammatory protein (MIP)-1α; MIP-1β; and regulated upon activation, normal T-cell expressed, and secreted (RANTES); tumor necrosis factor (TNF)-α. Luminex analyses were performed according to the manufacturer’s protocol, with minor modifications.

Briefly, samples were diluted 1:4, and recombinant cytokines were reconstituted and serially diluted to make a low-PMT standard curve. Beads with capturing antibodies were combined with standards and samples and added to a 96-well microtiter plate (Millipore Corporation, Billerica, USA) and incubated for 2 h before adding detection antibody and streptavidin-PE (Bio-Rad, Hercules, USA). The plate was analyzed with a Bio-Plex 200 System Workstation. Test runs were performed before analyses of the included samples to optimize the Luminex analyses. All samples were analyzed in duplicate to enhance precision.

### In vitro capillary-like tube formation assay

Human umbilical vein endothelial cells (HUVECs) were harvested and seeded onto a Matrigel surface (Corning Inc., Corning, USA) at 3 × 10^4^ cells/per well in 48-well plates and then incubated in DMEM for 6 h. Tube formation was observed under a phase-contrast microscope and photographed. Tube formation ability was quantified by counting the total tube length in three randomly chosen microscopic fields per well under ×40 magnification. The results were expressed as the mean tube length and normalized to the control. The experiment was repeated three times.

### Statistical analysis

Data are presented as the mean ± SEM. The statistical significance of differences was determined by unpaired Student’s *t*-tests to compare two means or one- or two-way ANOVA analysis followed by Tukey’s post hoc test or Sidak’s multiple-comparison test for repeated measurements or multiple comparisons. All statistical analyses were performed using GraphPad Prism 6.0 software (La Jolla, USA). *P* < 0.05 was considered statistically significant.

## Results

### Characteristics of short and prolonged myocardial I/R

**Fig. 1:**
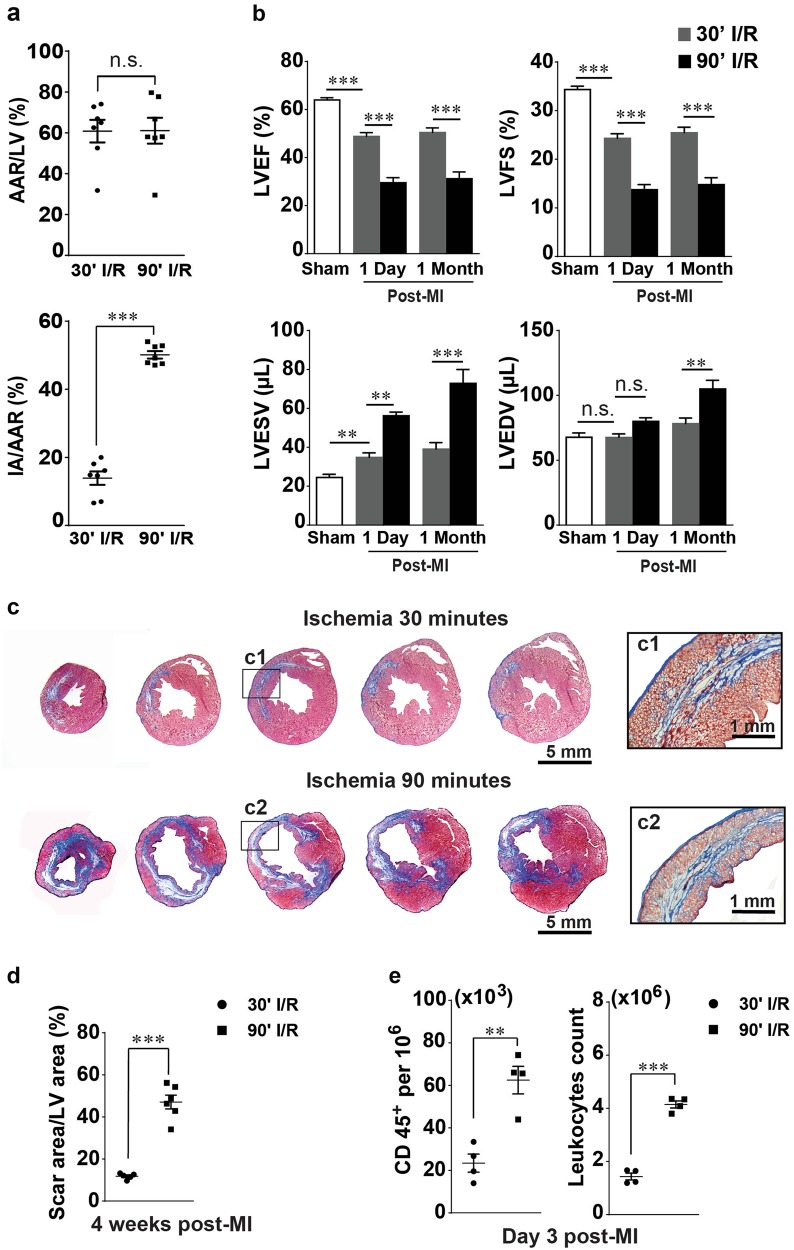
Comparison of myocardial injury and inflammatory cell infiltration during reperfusion following 30 and 90 min of ischemia. **a** Assessment of infarction size by Evans Blue and TTC shows equal areas at risk (AARs) but unequal infarcted areas (IAs) after 30 and 90 min of ischemia followed by 24 h of reperfusion (*n* = 6–7). **b** Results of echocardiographic assessment of the mice that underwent 30 or 90 min of I/R at day 1 and day 28 after MI (*n* = 5–6). **c** Representative images of Masson’s trichrome staining of the infarcted hearts at 28 days after MI. **d** Average scar area on post-MI day 28 (*n* = 5–6). **e** Leukocyte (CD45^+^) infiltration of infarcted hearts following 30 or 90 min of I/R on day 3 after MI (*n* = 4). Data are shown as the mean ± SE; n.s. means no significance, **P < 0.01, ***P < 0.001 as indicated

To characterize the myocardial injury induced by reperfusion following 90 min of ischemia, we compared myocardial salvage and cardiac performance after 30 and 90 min of ischemia in the closed-chest mouse model of myocardial I/R. Assessment of the area at risk (AAR) after 24 h of reperfusion revealed no difference between 30 and 90 min of ischemia, while the infarcted area (IA)/AAR significantly increased in 90 min of ischemia compared with 30 min of ischemia (Fig. 1a). Consistently, cardiac performance after 30 min of ischemia was worsened, while 90 min of ischemia caused a much more severe functional decline with significant alterations of LVEF and LVFS, as well as excessive LV chamber dilatation characterized by enlarged LVEDV and LVESV (Fig. 1b). Moreover, histological analysis at 28 days after MI revealed a considerably thinned LV wall with a reduced amount of myocardium in the AAR and conspicuous enlargement of LV chamber after 90 min of ischemia in comparison with 30 min of ischemia (Fig. 1c). The scar area in the 90-minute ischemia group was ~3 times larger than that in the 30-min ischemia group (Fig. 1d). In addition, flow cytometric analysis at day 3 after MI showed a significant difference in inflammatory cell influx between 30 and 90 min of I/R: there were 3 times more CD45^+^ cells detected in the infarcted hearts after 90 min of ischemia than after 30 min of ischemia (Fig. 1e). These data indicate that reperfusion following 90 min of myocardial ischemia causes much more severe cardiomyocyte loss, cardiac dysfunction, and inflammatory influx than reperfusion following 30 min of ischemia; therefore, intervention at 90 min of ischemia should be considered as late reperfusion. Therefore, we used 90 min I/R in our following studies as a prolonged ischemia to imitate late reperfusion.

### Temporal dynamics of the cellular component of the immune response in the heart following prolonged myocardial I/R

**Fig. 2:**
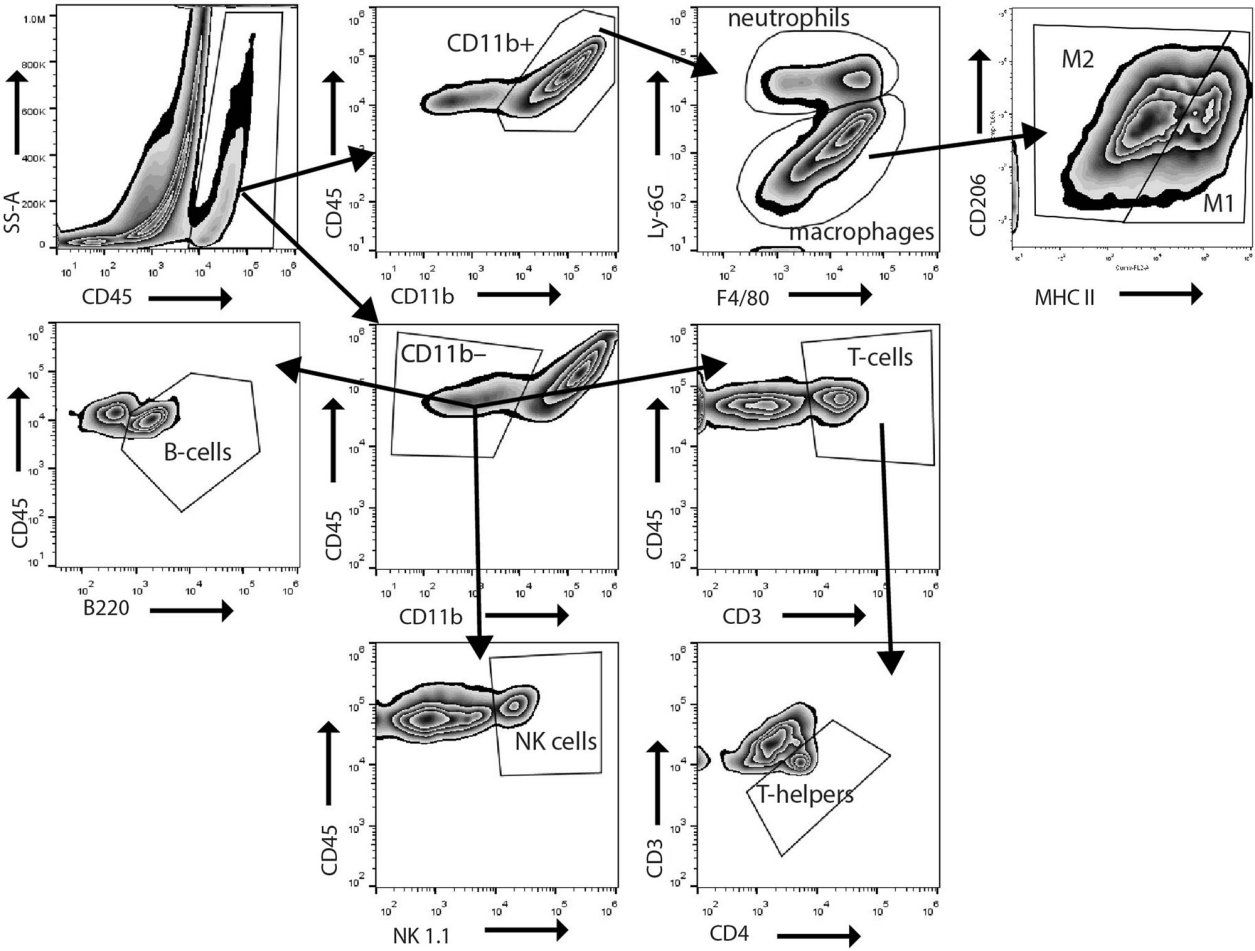
Gating strategy for identification of immune cells isolated from mouse hearts. Single-cell suspensions from digested hearts were analyzed for CD45 and CD11b expression. CD45^+^CD11b^+^ myeloid cells were divided into F4/80^+^ macrophages and Ly-6G^+^ neutrophils. The F4/80^+^ macrophages were further divided based on M1 (CD206^low^, MHC class II^high^) or M2 (CD206^high^, MHC class II^low^) polarity. CD45^+^CD11b^−^ (nonmyeloid) cells comprised T-cells (CD3^+^), B-cells (B220^+^), and natural killer (NK) cells (NK1.1^+^). T-cells were further examined for the T-helper subfraction (CD3^+^CD4^+^)

Next, we analyzed the dynamics of cellular components of the immune response after prolonged I/R in hearts. Immune cell populations extracted from the sham and prolonged I/R hearts were analyzed by flow cytometry using the strategy indicated in Fig. 2. Single-cell suspensions from digested hearts were analyzed for CD45 and CD11b expression. CD45^+^CD11b^+^ myeloid cells were divided into F4/80^+^ macrophages and Ly-6G^+^ neutrophils, with the F4/80^+^ macrophages further divided into M1 (CD206^low^, MHC class II^high^) and M2 (CD206^high^, MHC class II^low^) cells. CD45^+^CD11b^−^ (nonmyeloid) cells comprised T-cells (CD3^+^), B-cells (B220^+^), and natural killer (NK) cells (NK1.1^+^). T-cells were further examined for the T-helper subfraction (CD3^+^CD4^+^).

**Fig. 3:**
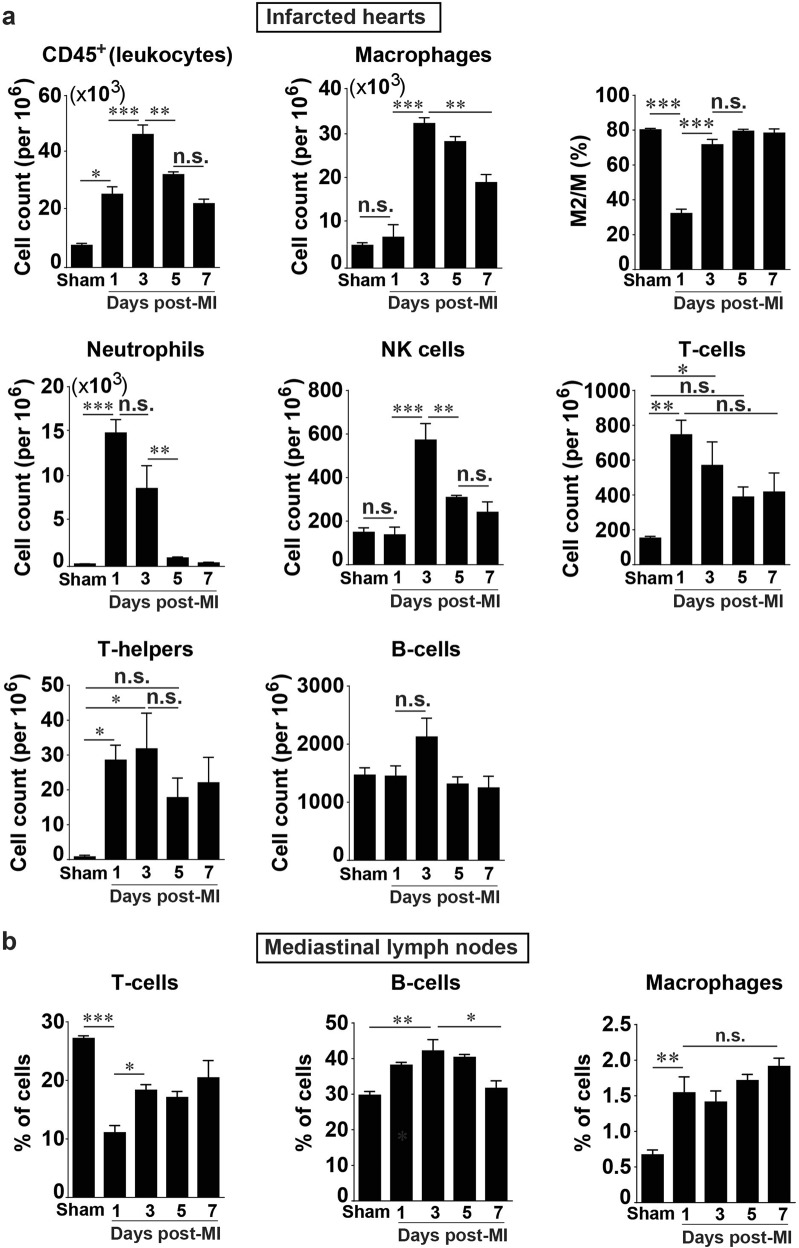
Quantification and characterization of the temporal dynamics of immune cells in the heart and mediastinal lymph nodes (MLNs) after prolonged myocardial I/R. **a** Time course of post-MI changes in the heart (*n* = 4). **b** Post-MI dynamics of major immune cell subsets in MLNs. The Y-axis shows percentages of the cell population among 5 × 10^4^ cells from mouse MLN analyzed by flow cytometry (*n* = 4). Data are shown as the mean ± SE; n.s. means no significance, *P < 0.05, **P < 0.01, ***P < 0.001 as indicated

Flow cytometric analysis revealed the influx of all major leukocyte populations after the onset of prolonged I/R (Fig. 3a). Leukocytes (CD45^+^ cells) gradually increased in number after prolonged I/R, peaked on day 3 after infarction, and dissipated afterwards (Fig. 3a, upper panel). Neutrophils were one of the first cell types recruited to the site of infarction and peaked on day 1 after MI, with a more than 50-fold increase compared with that in the sham operated hearts (Fig. 3a, middle left panel). Despite such remarkable neutrophil accumulation, macrophages were still the most abundant cell type after infarction, reaching a peak on day 3 after MI (Fig. 3a, upper middle panel). Further analysis revealed that in the resting state, the vast majority of macrophages in the heart were M2-like macrophages, which were significantly reduced on day 1 after MI, then recovered to a level similar to that seen in the sham group on day 3 after MI and remained stable up to day 7 after MI (Fig. 3a, upper right panel). The NK cell content in the infarcted hearts did not differ from that of the sham-operated hears on day 1 after MI, but it significantly increased and reached a peak at day 3 after MI (Fig. 3a, middle panel).

T-cells as well as T-helper infiltration significantly increased on day 1 after MI in the infarcted hearts compared with the sham-operated hearts and remained high up to day 3 after MI but decreased to a level statistically indistinguishable from that in the sham hearts on subsequent days (Fig. 3a, middle right panel and lower left panel). B-cells had a slight, statistically nonsignificant increase on day 3 after MI in infarcted hearts compared with the sham hearts (Fig. 3a, lower right panel). These results demonstrate that the infiltration of immune cells during prolonged I/R occurs in a time-dependent manner and suggest complex interactions among these immune cells following myocardial I/R.

### Temporal dynamics of major cellular subsets in MLNs following prolonged myocardial I/R

It has been well documented that the adaptive immune system plays an important role in inflammation after infarction [6, 9, 11, 26]. There is evidence suggesting crosstalk between the heart and MLNs [27]. We thus compared simultaneous changes following myocardial I/R between the hearts and in the MLNs by isolating immune cells from the MLNs harvested along with the hearts and subjecting them to flow cytometric analysis. T-cells in the MLN were reduced by ~3-fold at day 1 after MI compared with those in the sham group, and they partially recovered during days 3–5 after MI but showed no difference with the sham group on day 7 after MI (Fig. 3b, left panel). B-cells were dominant over macrophages and T-cells in the MLNs; the number of B-cells had increased at day 1 after MI, reached a maximum at day 3 after MI, and then returned to the level seen in the sham group (Fig. 3b, middle panel). Noticeably, macrophages in the MLN were increased by ~ 2-fold on day 1 after MI and maintained at a higher level up to day 7 after MI (Fig. 3b, right panel). These data indicate that the cellular composition of MLNs undergoes significant changes following prolonged myocardial I/R.

### Temporal dynamics of cytokines and chemokines following prolonged I/R in the heart and serum

**Fig. 4:**
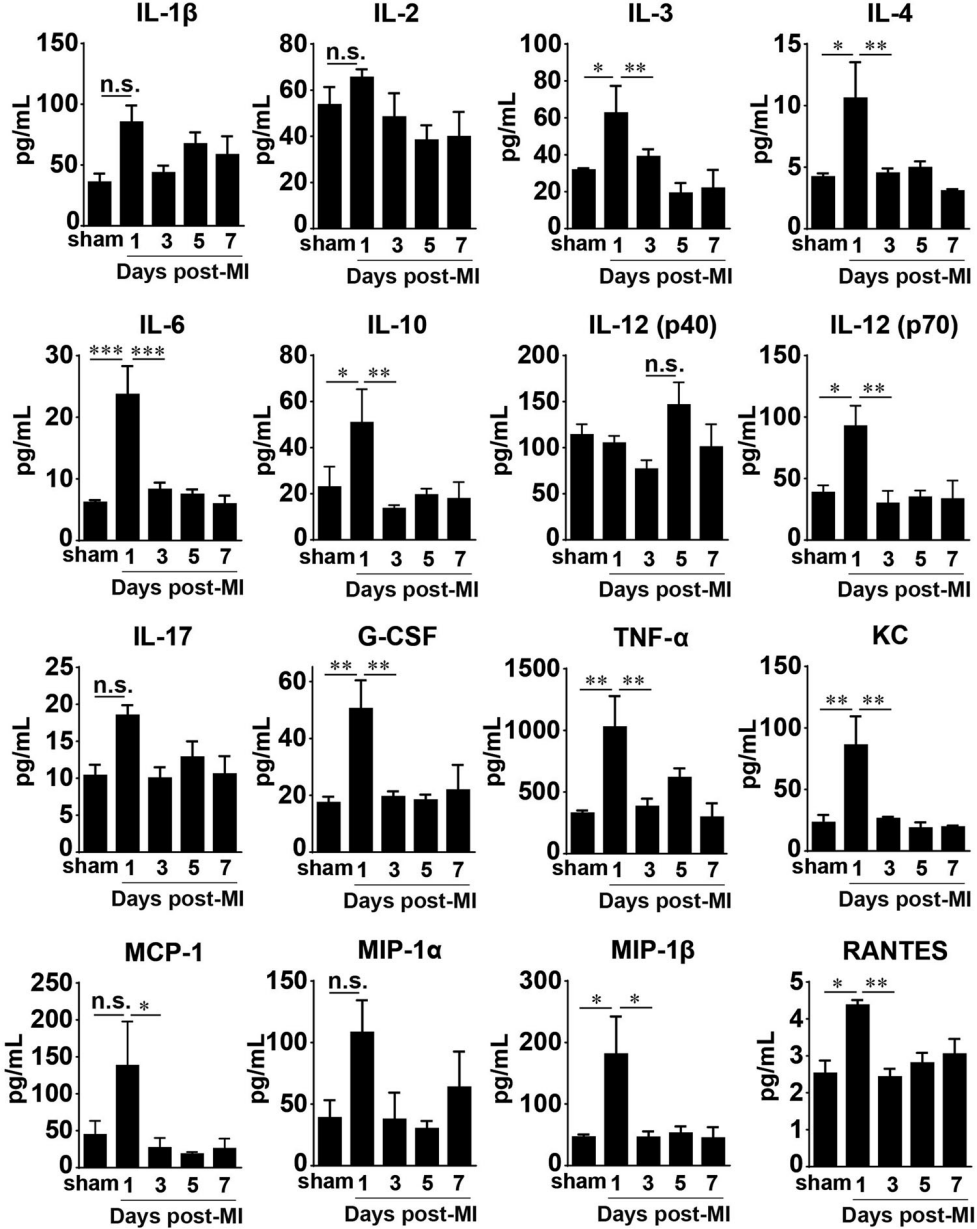
Serum cytokine and chemokine levels in mice after prolonged myocardial I/R. Cytokine and chemokine concentrations in serum were measured using a Bio-Plex assay (*n* = 4 for sham; *n* = 5–7 otherwise). Data are shown as the mean ± SE; n.s. means no significance, *P < 0.05, **P < 0.01, ***P < 0.001 as indicated

To obtain a picture of the molecular component of the immune response in prolonged ischemia reperfusion, we analyzed both the heart and the serum from the same mouse. The serum levels of the major cytokines and chemokines we examined were notably elevated only on day 1 after prolonged I/R, although 6 of the 16 shown in the figure did not display statistical significance compared with the adjacent time points and/or the sham group (Fig. 4). The most conspicuous changes were in the levels of TNF-α, G-CSF, IL-3, IL-4, IL-6, IL-10, IL-12p70, RANTES, KC, MCP-1, and MIP-1β. The others, namely, IL-1β, IL-2, IL-12p40, IL-17, and MIP-1α, had a visible but not statistically significant elevation compared with the sham group.

**Fig. 5:**
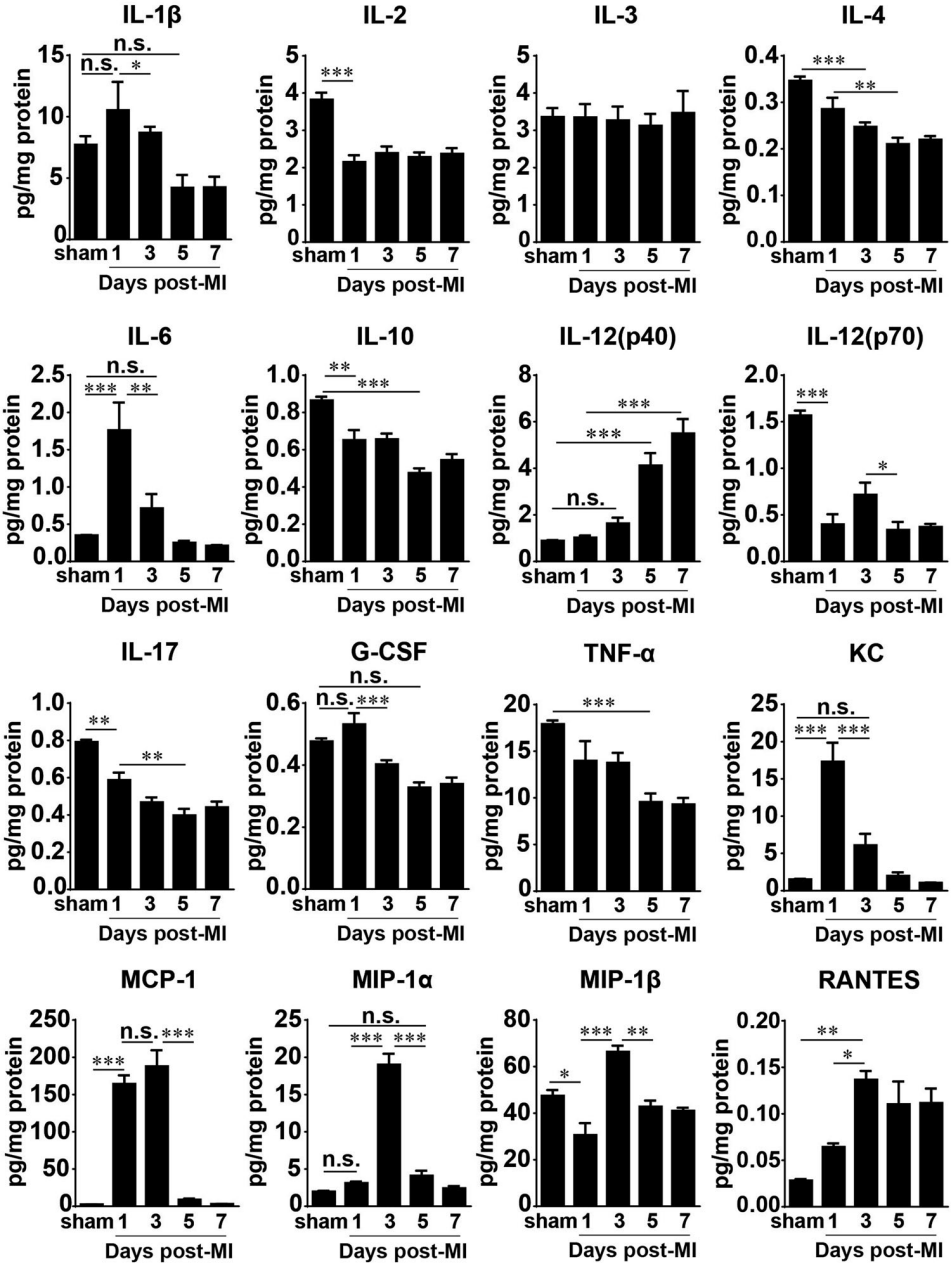
Cytokine and chemokine levels in infarcted hearts subjected to prolonged I/R. Cytokine and chemokine concentrations in the heart were measured using a Bio-Plex assay (*n* = 4 for sham; *n* = 5–7 otherwise). Data are shown as the mean ± SE; n.s. no significance, *P < 0.05, **P < 0.01, ***P < 0.001 as indicated

The levels of major cytokines in the infarcted hearts significantly changed in a time-dependent manner (Fig. 5), although IL-3 remained unchanged during the first 7 days after MI in the heart and was elevated in the serum only on day 1 (Fig. 4). IL-2, IL-10, IL-12 (p70), and IL-17 were significantly decreased at day 1 after MI and remained reduced compared with those in the sham group during the following days. The level of IL-4 in the heart significantly dropped at 3 days after MI, and the levels of IL-1β, G-CSF, and TNF-α were remarkably decreased starting at day 5 after MI. Conversely, the chemokines in the infarcted hearts showed different kinetics: the levels of KC (CXCL1) and MCP-1 increased by ~10-fold and ~60-fold, respectively, on day 1 after MI compared with those in the sham group; whereas KC had already decreased by day 3, MCP-1 remained at a high level on day 3 after MI. MIP-1α, MIP-1β, and RANTES levels were higher in the infarcted group than in the sham group, and they all peaked on day 3 after infarction but had different patterns. RANTES(CCL5) gradually increased, peaking on day 3, and remained at a high level up to day 7 after MI. MIP-1α or CCL3 was significantly increased only on day 3 after MI and not at the other time points examined, and MIP-1β (CCL4) was decreased at day 1 after MI and then increased at day 3 but returned to the sham level at days 5 and 7 after MI (Fig. 5).

### Effects of CSA on the outcome of prolonged myocardial I/R

**Fig. 6:**
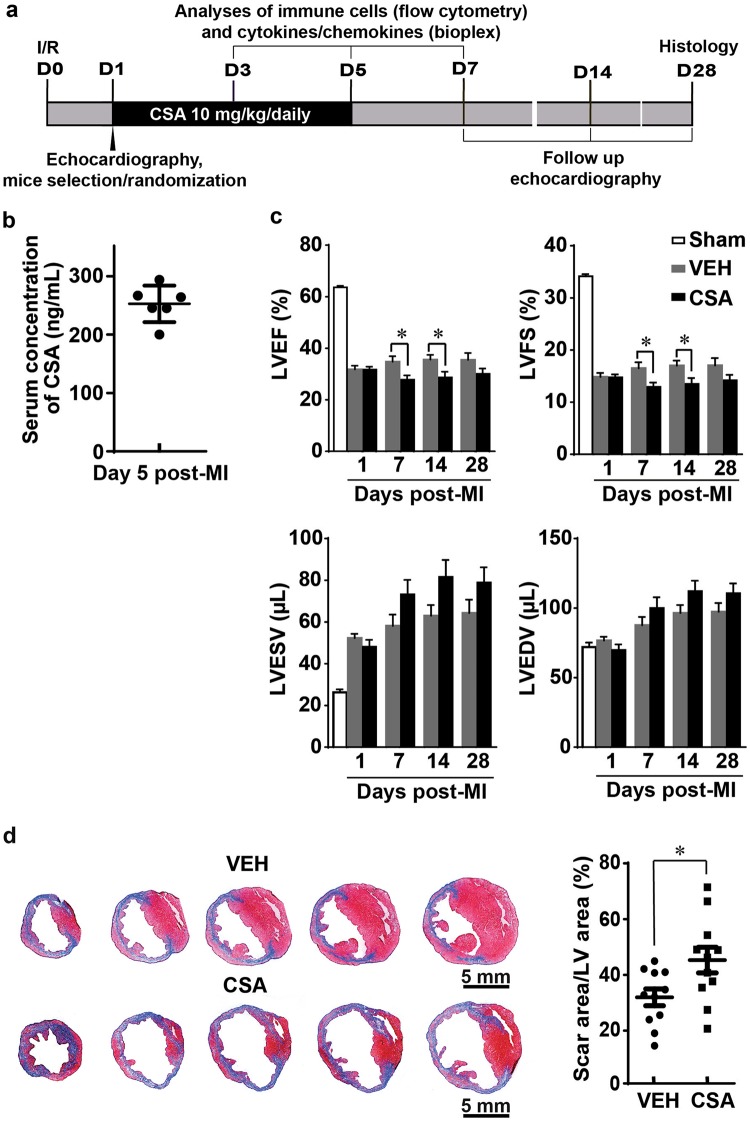
Effect of cyclosporine A (CSA) on the outcome of prolonged myocardial I/R. **a** Scheme of the experimental design. **b** CSA concentration in mouse serum during treatment (*n* = 6). **c** Comparison of echocardiographic parameters (LVEF, LVFS, LVESV, and LVEDV) between the VEH and CSA groups on the 7th and 14th days after MI (*n* = 11). **d** Representative images of Masson’s trichrome staining and average scar areas on post-MI day 28 (*n* = 11). VEH vehicle (control group), CSA cyclosporine A (treatment group). Data are shown as the mean ± SE; *P < 0.05 as indicated

Next, we moved forward to address the question of whether CSA affects the immune response following prolonged myocardial I/R. One day after induction of myocardial I/R, the mice were randomly assigned to two groups, namely, VEH and CSA, based on echocardiographic parameters. CSA was given daily from day 1–5 after MI as indicated in Fig. 6a. The average CSA concentration in mouse serum on day 5 after infarction was ~250 ng/ml (Fig. 6b). Echocardiographic analysis showed that cardiac performance at day 1 after MI was comparable between the VEH and CSA groups (Fig. 6c). However, the LVEF and LVFS became worse in the CSA group than in the VEH group on days 7 and 14 after MI, although they did not show a significant difference at day 28 after MI. In addition, the LVESV and LVEDV tended to increase over the post-MI time course in both the VEH and CSA groups, but higher values were observed in the CSA group than in the VEH group, though the difference was not statistically significant. These results indicate that heart function worsens after CSA treatment, which is associated with a tendency toward left ventricular dilatation. Consistently, histological examination of the mouse hearts 4 weeks after the infarction revealed a larger scar area with a thinner infarcted wall in the CSA group than in the VEH group (Fig. 6d). Taking these data together, we concluded that CSA administered in the acute phase of MI caused by prolonged myocardial I/R aggravates myocardial injury and remodeling.

### Effects of CSA on the immune response following prolonged myocardial I/R

**Fig. 7:**
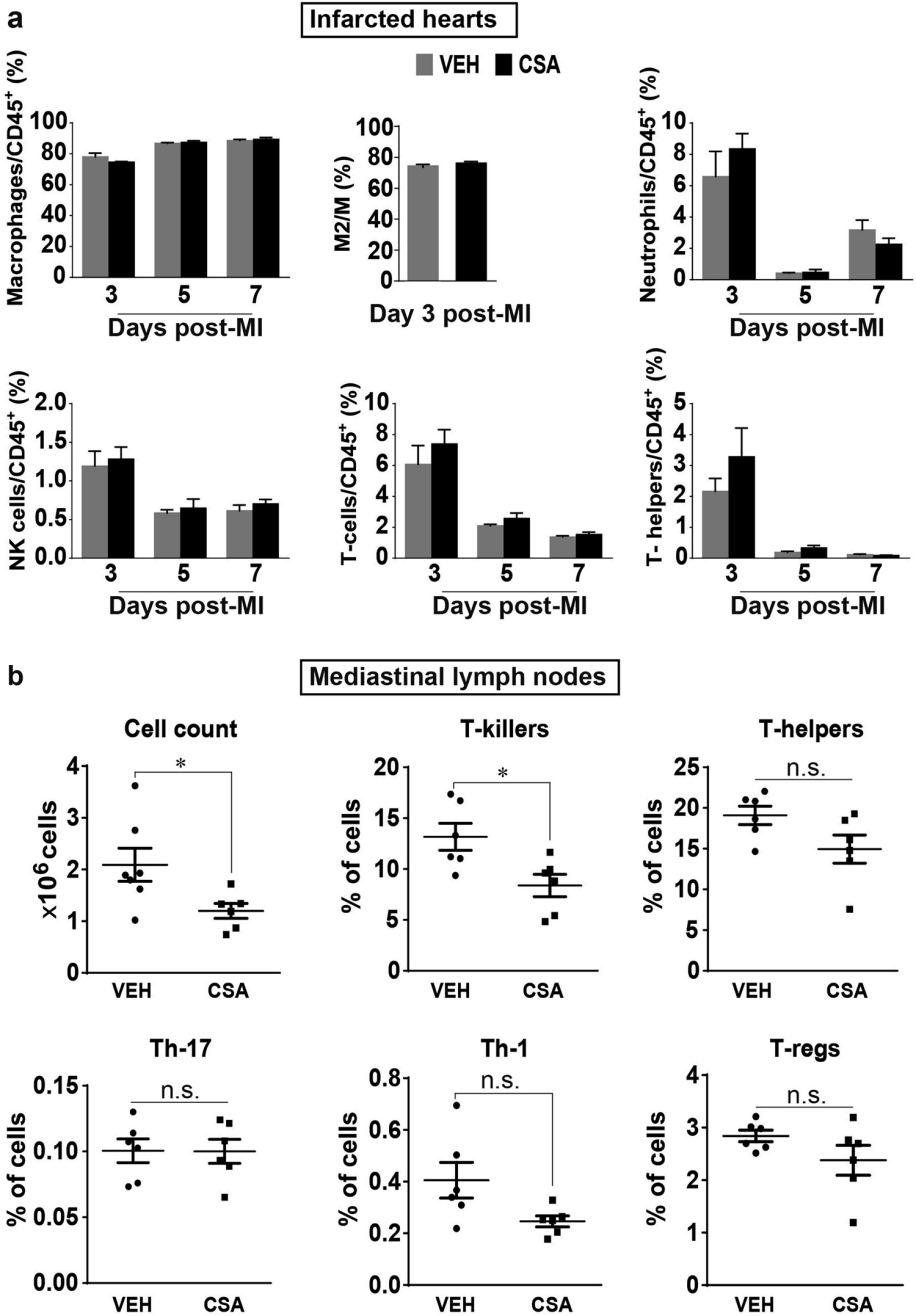
Effects of CSA on the number of immune cells in the infarcted heart and MLNs in prolonged myocardial I/R. **a** Comparison of the changes in the infarcted heart. The Y-axis shows the ratio (%) of cells of interest to CD45^+^ cells from the heart of the same mouse subjected to flow cytometry analysis (*n* = 5–7). **b** Comparison of the changes in MLNs. The Y-axis (except in the first graph) shows the percentage of the chosen cell population among 1.5 × 10^5^ cells from mouse MLNs analyzed by flow cytometry (*n* = 6). VEH vehicle (control group), CSA cyclosporine A (treatment group). Data are shown as the mean ± SE; n.s. no significance, *P < 0.05 as indicated

To determine the effect of CSA on the immune response following prolonged myocardial I/R, we conducted the same sets of experiments that were described in the previous sections regarding the immune response in mice after MI, but with the comparison between the VEH and CSA groups (Fig. 7). The total number of lymphocytes (CD45^+^) was used as an internal control; thus, the ratio of the cells of interest to total lymphocytes was used to represent the data. Because pharmacological intervention with CSA began at day 1 after infarction, the first time point at which we made a comparison was day 3 after MI. There were no significant differences in the ratios of different inflammatory cells, including neutrophils, macrophages, NK-cells, T-cells, and T-helper cells, between the VEH and CSA groups on days 3–7 after MI or in the M2/M ratio at day 3 after MI (Fig. 7a). These data suggest that CSA has no profound effects on the major cellular components of the immune response when given from day 1 after MI.

At the same time, CSA significantly reduced the total cell count (Fig. 7b) and the number of T-killer cells but not T-helper cells in the MLNs of the same infarcted mice at day 5 after MI (Fig. 7b), suggesting that CSA has a significant effect on the cellular content of MLN.

**Fig. 8:**
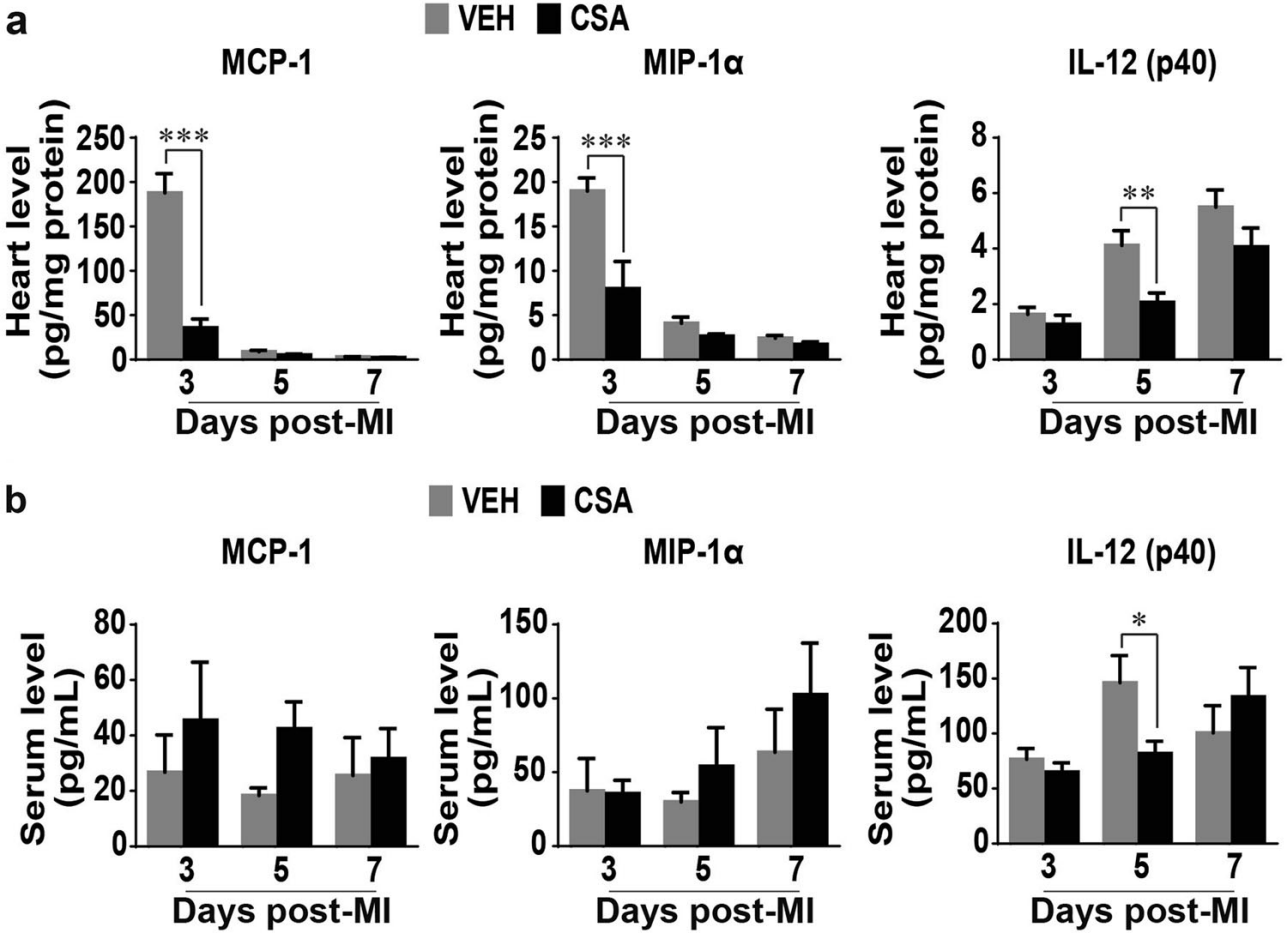
Effects of CSA on the serum and heart levels of cytokines and chemokines involved in the immune response following prolonged myocardial I/R. **a** Changes in the heart (*n* = 5–7). **b** Changes in the serum (*n* = 5–7). VEH vehicle (control group), CSA cyclosporine A (treatment group). Data are shown as the mean ± SE; *P < 0.05, **P < 0.01, ***P < 0.001

Further analysis of the molecular component of the immune response in the infarcted hearts showed that CSA significantly reduced the levels of proinflammatory MCP-1 and MIP-1α on day 3 after MI and IL-12 (p40) on day 5 after MI in the infarcted hearts (Fig. 8a). However, CSA did not affect the serum levels of MCP-1 or MIP-1α and significantly reduced the IL-12 (p40) level only on day 5 after MI (Fig. 8b).

### CSA administration following prolonged myocardial I/R is associated with decreased vascularity in the infarcted heart

**Fig. 9:**
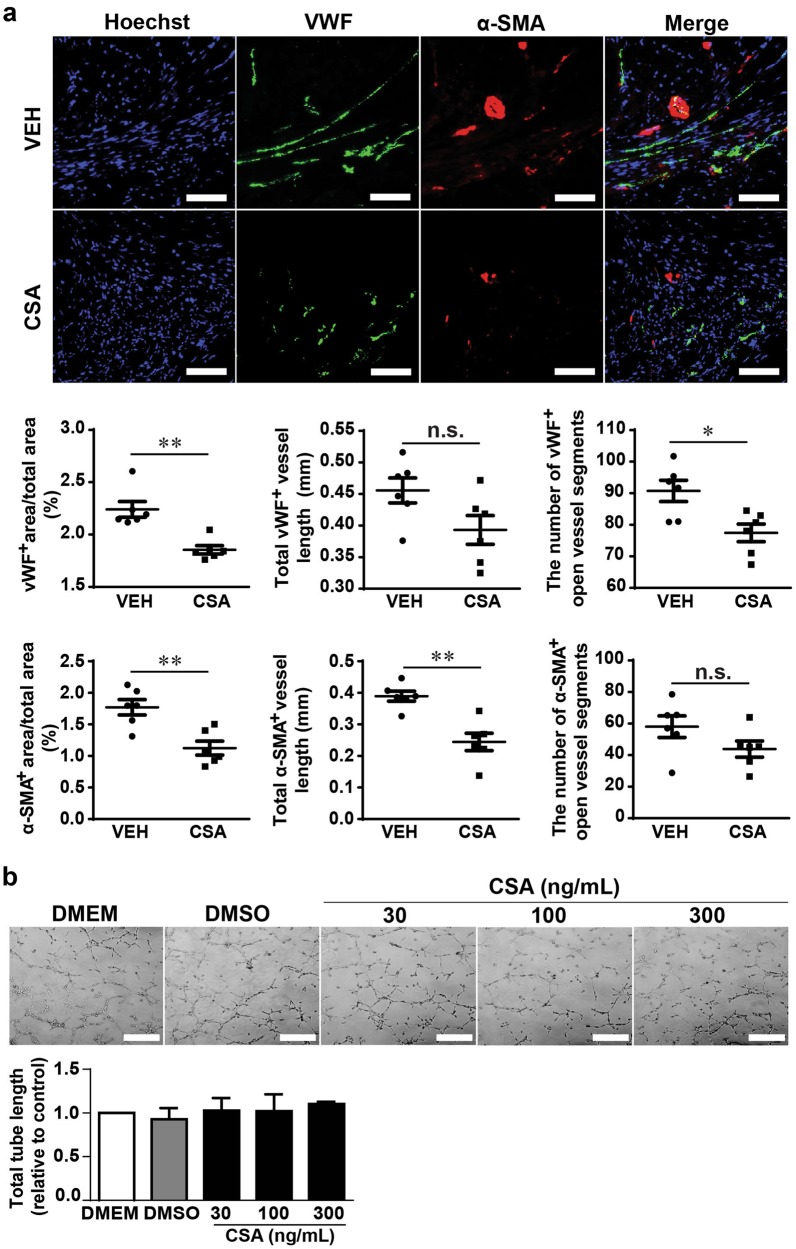
Effects of CSA on the vascularization of the heart after prolonged myocardial I/R and HUVEC tube formation. **a** Representative images and average analysis results of infarcted-zone and border-zone vascularization 4 weeks after MI in the VEH and CSA groups. Vascularization was assessed in 3 sections of each heart (*n* = 6). Scale bar = 50 μm. **b** Representative images and average analysis results of capillary-like tube formation by human umbilical vein endothelial cells (HUVECs) after CSA 30, 100, 300 ng/ml treatments for 6 h (summarized data from 3 replicates). Scale bar = 100 μm. VEH vehicle (control group), CSA cyclosporine A (treatment group), vWF von Willebrand factor, α-SMA α-smooth muscle actin. Data are shown as the mean ± SE; n.s. means no significance, *P < 0.05, **P < 0.01 as indicated

Because MCP-1 is a potent stimulator of angiogenesis in infarcted hearts [28, 29] and IL-12 is believed to possess antiangiogenic effects [30], we next examined whether CSA affects angiogenesis after MI by comparing the vascularization of infarcted hearts in the VEH and CSA groups. The vessel density, total vessel length, and number of open vessel segments, reflected by vWF as a marker of endothelial cells and α-SMA as a marker of smooth muscle cells, in the infarct and border zones on day 28 after MI were significantly lower in the CSA group than in the VEH group (Fig. 9a). Notably, the vWF^+^ area, vessel length and number of open vessel segments in the remote zone were comparable between the VEH and CSA groups, while the α-SMA^+^ area and vessel length but not the number of open vessel segments were significantly lesser than those in the VEH group (Supplemental Fig. 1), indicating the different pattern of vascularization between the infarcted/border and remote zones.

Next, to exclude the possibility of a direct effect of CSA on endothelial cells, we used a capillary-like tube formation assay to evaluate the impact of CSA on in vitro angiogenesis. CSA administered at concentrations of 30, 100, and 300 ng/ml (the average concentration of CSA in mouse serum was ~250 ng/mL) did not influence the ability of HUVECs to form tubes spontaneously (Fig. 9b), suggesting that the reduced vascularization of CSA-treated infarcted hearts is not due to the direct effect of CSA on endothelial cells.

## Discussion

The present study demonstrates that the peak levels of cytokines/chemokines in systemic circulation after MI caused by prolonged myocardial I/R are synchronized with the maximal influx of neutrophils and T-cells in the infarcted heart. In turn, the peak of local cytokines/chemokines in the infarcted heart coincides with the macrophage and NK cell infiltration of the heart. These findings reveal a previously unrecognized pattern of immune response in prolonged myocardial I/R, enable tracing of different stages of immune cell recruitment to the infarcted site and detection of the infarction stage through the measurement of corresponding cytokines/chemokines in serum. In addition, we are the first to describe the interference of CSA with the immune response following prolonged myocardial I/R. This finding provides new knowledge on the interaction of CSA with infarcted hearts and suggests that the caution might be taken when use of CSA in the acute phase of MI with or without the cell transplantation.

### Immune modulating power of late myocardial reperfusion

One novelty here is the description of the temporal dynamics of immune cell infiltration and dissipation following MI caused by prolonged myocardial I/R. Interestingly, the overall dynamics of the immune response as well as the peaks and temporal dynamics of neutrophils, macrophages, NK-cells, T-cells, and T-helpers demonstrated in our study for prolonged ischemia are identical to those described for short myocardial I/R by Yan et al. [11]. However, we found that prolonged ischemia causes an immune response several times the magnitude of the immune response following short I/R (Fig. 1e). These findings indicate that the general immune response following myocardial I/R has a consistent velocity even in a much larger infarction after prolonged ischemia. However, the immune response caused by permanent myocardial ischemia in MI is notably delayed [11, 22]. This, in turn, confirms that the patterns of immune response are different in the myocardial I/R and permanent ligation models. Hypothetically, reperfusion opens the routes for inflammatory cells to and from the site of injury and therefore promotes faster immune response and subsequently faster healing [3]. The identical dynamics of immune response in brief and prolonged I/R, data on delayed immune response in permanent ischemia [11, 22], and evidence that late reperfusion has a more favorable outcome than ischemia [2–5] suggest that late reperfusion promotes a remarkable modulatory effect on the immune response after MI. In this respect, knowledge of the normal immune response following prolonged ischemia is essential for molecular cardiology. Further study to determine the reperfusion time window for the optimal modulation of the immune response after MI is required. Moreover, it is worth comparing the ratio and number of various immune cells following short and prolonged myocardial I/R in the same set of experiments.

### Macrophage behavior in the immune response following prolonged myocardial I/R

Our findings suggest that macrophages are numerically the dominant cell type recruited during the immune response to infarcted myocardium following prolonged I/R. Interestingly, there was no significant increase in macrophages in the heart in the first 24 h after the onset of prolonged ischemia, but subsequently there was a massive macrophage influx, with a peak at day 3 after infarction. In a study by Yan et al. [11], 30 min of ischemia also caused a peak in macrophage infiltration on day 3, but there was a statistically significant increase in macrophage infiltration on day 1 after MI as well. This discrepancy may be related to the different models of myocardial I/R used, i.e., the open-chest short I/R model in Yan’s study [11] versus the closed-chest prolonged I/R model in our study. In turn, permanent occlusion of the LAD artery in mice causes macrophage peak infiltration on days 4–5 after MI [11, 22], also indicating that macrophage infiltration occurs more quickly in the I/R model than in permanent LAD artery occlusion.

Analysis of CD206 (the mannose receptor) – a surrogate marker of M2-like macrophages – reveals that in the resting state, the vast majority of heart macrophages are CD206^high^. This is consistent with the fact that resident cardiac macrophages present in the heart at steady state [31, 32] and the fact that M2 is a default state for cardiac resident macrophages [11, 31, 32]. Furthermore, we found that on day 1 after prolonged ischemia, the vast majority of heart macrophages become CD206^low^, but, as mentioned above, this occurred without a notable increase in macrophage number. This is supported by a study showing excessive death and almost complete disappearance of heart resident macrophages in the first 24 h after ischemia [33]. In our study, apparent macrophage influx starts later than the first day after MI and is synchronized with the increase in the macrophage M2/M ratio. Three days after infarction, when the immune response reaches its peak, most of the macrophages express CD206. Since that time, the M2/M ratio returns to its steady state value and remains constant despite extant macrophage abundance. Eventually, our data reveal that M2 in the heart is the predominant phenotype of macrophages both in the steady state and 3 days after MI caused by prolonged myocardial I/R. However, it is impossible to reach any conclusion regarding macrophage functional switching based only on surface markers, and in this respect, our findings have serious limitations.

### Major molecular regulators of immune response after prolonged myocardial I/R

Another novelty of the current study resides in the comprehensive cytokine/chemokine analysis. There are countless reports on the involvement of a variety of cytokines and chemokines in the immune response after MI [11, 34]. However, a majority of studies have focused on the mRNA expression levels of the corresponding cytokines either in the serum or in the heart. Few studies have provided simultaneous assessments of cytokine protein levels in serum and in the heart. Regulation of the immune response after MI is extremely complex. However, many cytokines involved in this process are not necessarily its main orchestrators. Therefore, serum screening after prolonged myocardial I/R revealed that the majority of investigated cytokines and chemokines were elevated, peaking 24 h after infarction. In this regard, it is likely that the reaction of the serum represents a systemic response to MI, one that is nonspecific and includes cross-activation of multiple organs and systems, including the liver [35], the bone marrow [36], the spleen and other lymphoid organs [37], the kidney [38] and others. Our data show that the systemic response ceases quickly and that a further inflammatory response is driven locally at the site of infarction. In contrast to the serum, the cytokine/chemokine response in the heart has a specific pattern that is probably determined by heart infiltration with distinct immune cells. Thus, according to our data, only a few cytokines and chemokines discussed below should be considered specific regulators of the immune response after prolonged myocardial I/R.

IL-6 and IL-12p40 demonstrated an exceptional increase after prolonged myocardial I/R in comparison to the other cytokines. IL-6 is a potent metabolic and immune regulator that has different actions in multiple cell types, acting as both a proinflammatory cytokine and an anti-inflammatory myokine [39]. Moreover, activation of inflammation stimulates cardiomyocyte proliferation in the neonatal heart, and blockade of IL-6 or signal transducer and activator of transcription 3 (STAT3), its downstream effector, blocks heart regeneration after apical resection [40]. This makes IL-6 a possible candidate for an intermediary between systemic and local responses, as well as between the metabolic and inflammatory pathways, suggesting that IL-6 acts as a key initiator and regulator of metabolic and immune responses in MI [39, 41].

IL-12p40 is another well-known cytokine that is able to induce immune responses and has antiangiogenic activity. Deficiency of IL-12p40 improves cardiac repair after MI by promoting angiogenesis [30]. Our data show that IL-12p40 operates in later stages of inflammation starting on day 3 after MI. On the other hand, distinguishable neoangiogenesis in the infarcted heart starts only 3–4 days after infarction onset in mice when the inflammatory phase switches to the proliferative one [42]. Therefore, our data suggest that the timing of IL-12p40 secretion coincides with neoangiogenesis in the heart after prolonged I/R. It is possible that IL-12p40 is necessary to counterbalance the wide range of proangiogenic factors. Nevertheless, its function should be further clarified.

Levels of monocyte chemoattractant protein-1 (MCP-1, or CCL2) are significantly elevated in the heart from day 1 to 3 post-infarction. Moreover, its maximum levels in the heart coincide with massive macrophage infiltration. MCP-1 is a critical factor for the recruitment of monocytes/macrophages in the infarcted heart [28], and they are found to be the major source of MCP-1 [43]. MCP-1 is a potent inducer of angiogenesis [28, 29, 44] and plays an important role in promoting inflammatory tissue healing in an MIP-1α-dependent pathway [45]. Our findings showed that MIP-1α (CCL3) and MIP-1β (CCL4) are both elevated in the short term, beginning on day 3 after MI. Whether MIP-1α/β is downstream of MCP-1 in myocardial I/R requires further clarification.

Finally, based on our data, KC (CXCL1) and RANTES (CCL5) might also be considered important regulators of the immune response following prolonged myocardial I/R, as KC is significantly elevated in the serum and infarcted heart, peaking on day 1 after infarction. KC has been recognized to play a critical role in the induction of systemic inflammation and tissue damage after trauma-hemorrhage [46]. RANTES exhibits a consistent increasing trend beginning soon after infarction, with a maximum on day 3 after MI. RANTES plays an important role in determing infarct size and postinfarction heart failure in mice [47] and has prognostic value in humans [48].

### CSA administered in the acute phase of MI alters critical cytokines involved in the immune response and worsens the outcome of MI

Another notable finding in this study is that continuous administration of CSA in the acute phase of MI causes functional deterioration and adverse remodeling of infarcted mouse hearts in association with reduced vascularization. This finding runs counter to a substantial number of previous studies suggesting a protective role of CSA in MI [16, 17, 49]. This discrepancy may result from (i) different myocardial I/R models and (ii) different protocols of CSA administration between our study and others. Most studies on cardioprotection by CSA in mice are conducted using brief myocardial I/R [50, 51], while the prolonged I/R model employed in the present study induces massive cardiomyocyte loss and serious impairment of heart function (Fig. 1). In addition, CSA is administered during ischemia or immediately on reperfusion in the majority of studies on cardioprotection [50–52], whereas, in our study, CSA is administered from day 1 after MI, following echocardiographic assessment and proper randomization. Additionally, only a single injection of CSA is used in the majority of studies on cardioprotection by CSA [52], whereas we used continuous administration of CSA for 5 days to imitate immunosuppression similar to that used for nonautologous stem cell transplantation [12], though the single dose of CSA used in our study is comparable to that used in cardioprotective studies [52]. Our findings suggest that the negative effect of CSA on the infarcted heart might interfere with the benefits of stem cell transplantation when CSA is used along with cell transplantation in the acute phase of MI. The precise mechanism responsible for the negative effect of CSA on the infarcted heart needs to be explored in the future.

Surprisingly, administration of CSA does not significantly affect the cellular component of the immune response in the heart following prolonged I/R, despite the clear effect of CSA on cells from MLNs. This suggests that CSA administered from day 1 after infarction does not significantly influence the recruitment of immune cells to the infarction site. At the same time, we found that CSA causes a remarkable reduction in the levels of MCP-1, MIP-1α, and IL-12 in the infarcted heart following prolonged myocardial I/R. Given that the main producers of inflammatory cytokines and chemokines are immune cells, our findings imply that CSA changes the function of immune cells recruited to the infarction site; this may be the reason for the deterioration of infarcted hearts after CSA treatment. As noted above, CSA was given from day 1 after MI in this study. At this time, cytokine levels in serum as well as neutrophil and T-cell infiltration of the infarcted hearts are peaking. As long as this occurs before the first injection of CSA, CSA does not affect these events. Therefore, the effect of CSA on the earliest immune response caused by prolonged myocardial I/R needs to be elucidated in the future.

In addition, we found that CSA reduced the vascularization of infarcted hearts. The inability of CSA to impair HUVEC tube formation in vitro suggests that the reduction of postinfarction vascularization does not stem from the direct effect of CSA on endothelial cells. This implies, in turn, that reduced vascularization after CSA treatment may be caused at least in part by the suppression of MCP-1, MIP-1α, and IL-12. Given that MIP-1α is downstream of MCP-1 [45], underproduction of MCP-1 would provoke a decrease in MIP-1α as well. IL-12, in turn, is widely recognized as a suppressor of angiogenesis [30]. If MCP-1 induces angiogenesis, the reduction of MCP-1 by CSA might cause decreased angiogenesis after I/R. Under such conditions, less IL-12 is required to counterbalance MCP-1 and allow sufficient angiogenesis. However, these possibilities need to be confirmed by experimental data, and this is a limitation of the present study. Taking this into consideration, further study on the detailed mechanisms of CSA interference with immune response and angiogenesis after MI is highly desirable.

In conclusion, the current study is the first detailed description of the main temporal features of the immune response in the murine heart, systemic circulation, and MLNs caused by prolonged myocardial I/R. These findings reveal previously unrecognized fundamental aspects of the immune response following prolonged myocardial I/R. Such knowledge is essential for the development of novel approaches, including pharmacotherapies, for ischemic heart disease. Moreover, using the immune suppressor CSA, we demonstrate an intervention in the immune response after myocardial infarction caused by prolonged myocardial I/R and reveal that CSA deteriorates the infarcted heart, resulting in impaired heart function, increased infarct size, and reduced vascularity of the infarcted hearts. The latter may stem from the effect of CSA on the secretion of major cytokines involved in the immune response after infarction. These findings suggest that studies using CSA in the acute phase of MI need to be conducted with caution.

## Supplementary information

Supplemental Figure 1
