# Supplementary figures and images for: Temporal dynamics of immune response following prolonged myocardial ischemia/reperfusion with and without cyclosporine A
Source: Acta Pharmacol Sin. 2019 Mar 11;40(9):1168–83. doi: 10.1038/s41401-018-0197-1 (PMC6786364; doi:10.1038/s41401-018-0197-1)

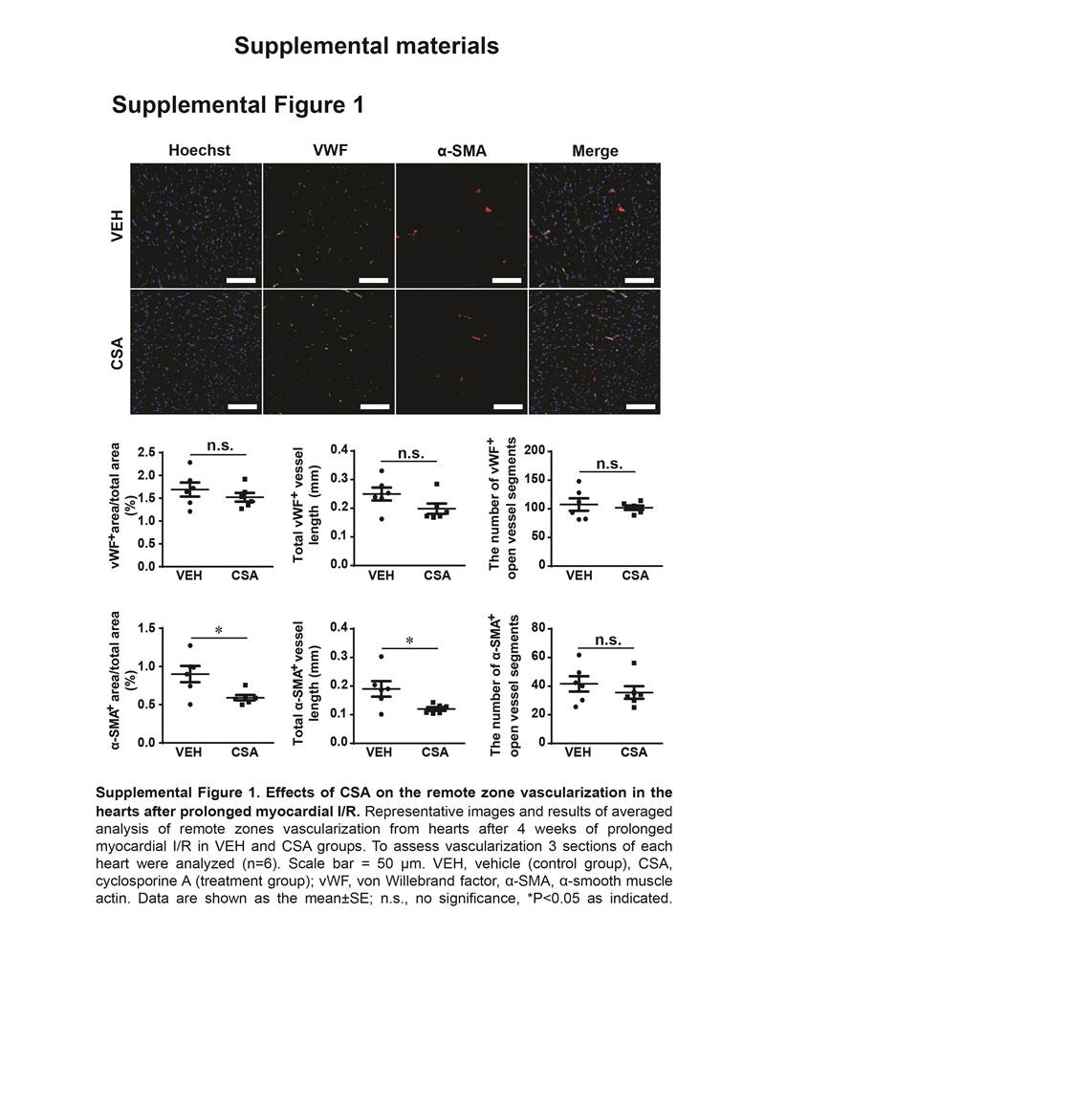

Supplement: Supplementary file 1 — Supplemental Figure 1 [file 41401_2018_197_MOESM1_ESM.png]
